# Transmissive Single-Pixel Microscopic Imaging through Scattering Media

**DOI:** 10.3390/s21082721

**Published:** 2021-04-13

**Authors:** Huaxia Deng, Guan Wang, Qiang Li, Qianzhen Sun, Mengchao Ma, Xiang Zhong

**Affiliations:** School of Instrument Science and Opto-Electronics Engineering, Hefei University of Technology, Hefei 230009, China; 2017110046@mail.hfut.edu.cn (G.W.); 2018110016@mail.hfut.edu.cn (Q.L.); 2018110013@mail.hfut.edu.cn (Q.S.); mmchao@hfut.edu.cn (M.M.); zhx0325@hfut.edu.cn (X.Z.)

**Keywords:** single-pixel imaging, microscopic imaging, multi-spectral, LC-SLM, scattering media

## Abstract

Microscopic imaging is of great significance for medical diagnosis. However, due to the strong scattering and absorption of tissue, the implementation of non-invasive microscopic imaging is very difficult. Traditional single-pixel microscopes, based on reflective optical systems, provide an alternative solution for scattering media imaging. Here, the single-pixel microscope with transmissive liquid crystal modulation is proposed. The microscopic ability of the proposed microscope is calibrated. The multi-spectral microscopic imaging of the object is demonstrated. The transmissive imaging of the object behind the scattering media is analyzed. The proposed prototype of the transmissive single-pixel microscope is expected to be applied in microscopic imaging through scattering media and medical imaging.

## 1. Introduction

Non-invasive microscopic imaging, a secure scatheless internal micro-objects imaging method, is pervasively applied in medical science, such as fundus examination [1,2,3,4,5], angiography [6], wound monitoring [7], and cancer nest detection [8,9]. These micro-objects have large scattering angles and much absorption to incident light [10,11,12]. Observing internal micro-objects requires methods with high penetration and low-light imaging capabilities, avoiding invasive observation of the microscopic structure within tissues. The penetration and scattering angles of incident light are both disparate in different wavelengths. Short wave incident light, such as X-ray, is efficient in improving the penetration and reducing scattering angle. Certain long wave incident light, such as infrared light, is absorbed enormously by cancer cells, and is suitable for multi-spectral imaging and locating cancer [13]. However, there is a certain compromise between deeper penetration and detecting cancer. As a result, non-invasive and multi-spectral imaging has limited capabilities in detection, which is critical in identifying cancer accurately.

Conventional microscopes, using visible light for illumination, have a penetration depth of only 10–20 μm, which are not suitable for non-invasive microscopic imaging. The confocal microscope, utilizing the light beam from focal plane for imaging, has up to 0.2 mm penetration depth [14,15,16]. Multi-photon fluorescence microscopy has the penetration of 0.5 to 1 mm by exciting fluorophore to produce the emitted light, without the ability of thick tissue imaging [17]. X-ray and γ-ray imaging use light sources with low scattering coefficients to reduce the scattering angle in the propagation path and can directly penetrate human tissues [18]; however, high-energy rays may cause irreparable damage to the human body. Traditional methods are limited to be employed in non-invasive multi-spectral imaging.

The deeper penetration and multi-spectral imaging of tissue are difficult to achieve simultaneously in traditional methods, which are both based on the theory of linear propagation of light and the theory of keyhole imaging [19,20,21,22,23,24,25,26]. Recent studies on single-pixel imaging show that the theory of linear propagation of light and the theory of keyhole imaging can be broken through, and object reconstruction can be performed based on one-dimensional data [27,28,29,30,31,32,33]. To reduce sampling data for single-pixel imaging, a set of modulated patterns is used to realize compressed sampling [34,35,36,37,38,39,40,41,42]. It improves the speed of single-pixel microscopic imaging. Single-pixel imaging provides a potential solution for penetrating scattering media. Penetrating scattering media are achieved through second-order correlation [43]. There are tests and reference optical paths for correlation. To simplify the optical path in the scattering media imaging, the Digital Mirror Device (DMD) is used in the optical path and the reference path is unnecessary [44]. The simplified optical system combined with microscopic technology is utilized to achieve 4× microscopic imaging through scattering media [45]. Then, the pattern codification is proposed to cancel the optical influence of DMD diamond-like shaped structure [46]. Besides, simultaneous infrared and visible light microscopic imaging are achieved by single-pixel microscopic imaging [47]. The single-pixel microscope, using a photodetector (PD) instead of a traditional array detector, has the superiority of having a wideband response and high sensitivity to the measured signal. Single-pixel imaging is suitable for overcoming interference and detecting weak light. However, the optical path of traditional single-pixel microscopic imaging is a reflective structure, in which the pattern is generated by irradiating the DMD with a light source, and then being reflected onto the object. The PD receives the light intensity signals modulated by the object, which are used to recover the image.

To achieve single-pixel microscopic imaging, we have proposed a single-pixel microscope that uses the liquid crystal spatial light modulator (LC-SLM) to modulate the object and Fourier Transform (FT) to realize reconstruction. Here, LC-SLM is used to set up a transmissive optical system. This transmissive system is simple and efficient, in which the number of optical components is simplified and the 4F Fourier filter is removed because the diffraction phenomenon is negligible. The optical distortion of the illumination pattern is acceptable when the optical imaging system is simplified. In the experiments, single-pixel microscopic imaging with different magnifications were performed, and the image resolution was up to 9 μm. Microscopic imaging experiments with different compression ratios were performed, in which 10% of data in the frequency domain was demonstrated to be sufficient to reconstruct the image. The multi-spectral microscopic images were obtained by illuminating liver cancer slices with different spectra. Finally, the proposed transmissive single-pixel microscopes capability was demonstrated via microscopic imaging through scattering media.

## 2. Materials and Methods

### 2.1. Methods of Single-Pixel Imaging

Single-pixel imaging, as an alternative method to traditional imaging based on the array detector, encodes the two-dimensional optical image into the sequence of one-dimensional signals. To decrease measurements in single-pixel imaging, different patterns such as random patterns, Hadamard patterns, and Fourier patterns are generated to modulate the object. Due to characters of high sensitivity to low-light, quick response to high-frequency signals, and detectable of wide-band light compared with the Charge Coupled Device (CCD) sensor, the PD is used as a detector to measure intensities of patterns transmitted by the object. As a result, single-pixel imaging has natural advantages in compressed sampling and multi-spectral imaging. Meanwhile, single-pixel imaging with Fourier patterns is helpful in compressed imaging and recovering high signal-to-noise ratio images.

Sinusoidal Fourier patterns are composed of $2^{n}$ pixels and are generated from the following expression:

(1) $$f_{\Phi }\left(x,y,f_{x},f_{y}\right)=\cos\left(\frac{f_{x}}{N}x+\frac{f_{y}}{N}y+\Phi \right)$$

$\frac{f_{x}}{N}$ and $\frac{f_{y}}{N}$ are two-dimensional discrete frequencies of the sinusoidal pattern. Φ is the phase of the sinusoidal pattern and $\Phi =0,\frac{\pi }{2},\pi ,\frac{3\pi }{2}$. The pattern focused on the object is: $f_{\Phi }^{\prime }\left(x,y\right)=a+bf_{\Phi }\left(x,y\right)$. *a* represents the influence of ambient light, and *b* represents the loss coefficient of the light beam. The object is modulated with four different phases: $D_{0},D_{\frac{\pi }{2}},D_{\frac{3\pi }{2}},D_{\pi }$, which are combined to reduce the interference of ambient light by the differential algorithm. The four-step phase-shifted modulation is conducted to sample the frequency data of the object. The process can be expressed mathematically as

(2) $$D_{0}-D_{\pi }+i\left(D_{\frac{\pi }{2}}-D_{\frac{3\pi }{2}}\right)=2bk\underset{\Omega }{\;\int \int \;}R\left(x,y\right)\left(A-iB\right)dxdy$$

The R(x,y) is the 2D transmittance distribution function of the object. The recovered image of the object is represented by the R(x,y) in the experiments.

(3) $$A=\cos(2\pi \left(\frac{f_{x}}{N}x+\frac{f_{y}}{N}y\right))$$

(4) $$B=\sin(2\pi \left(\frac{f_{x}}{N}x+\frac{f_{y}}{N}y\right))$$

*k* is the light loss coefficient. After sampling the frequency data, the image of the object is recovered through inverse Fourier transform, as is shown below mathematically:

(5) $$R\left(x,y\right)=F^{-1}\left(D_{0}-D_{\pi }+i\left(D_{\frac{\pi }{2}}-D_{\frac{3\pi }{2}}\right)\right)$$

(6) $$R\left(x,y\right)=\frac{1}{2bk}\sum_{f_{x}=0}^{N-1}\sum_{f_{y}=0}^{N-1}\left(D_{0}-D_{\pi }+i\left(D_{\frac{\pi }{2}}-D_{\frac{3\pi }{2}}\right)\right)e^{-2\pi i(\frac{f_{x}}{N}x+\frac{f_{y}}{N}y)}$$

### 2.2. Transmissive Optical Modulation

**Figure 1:**
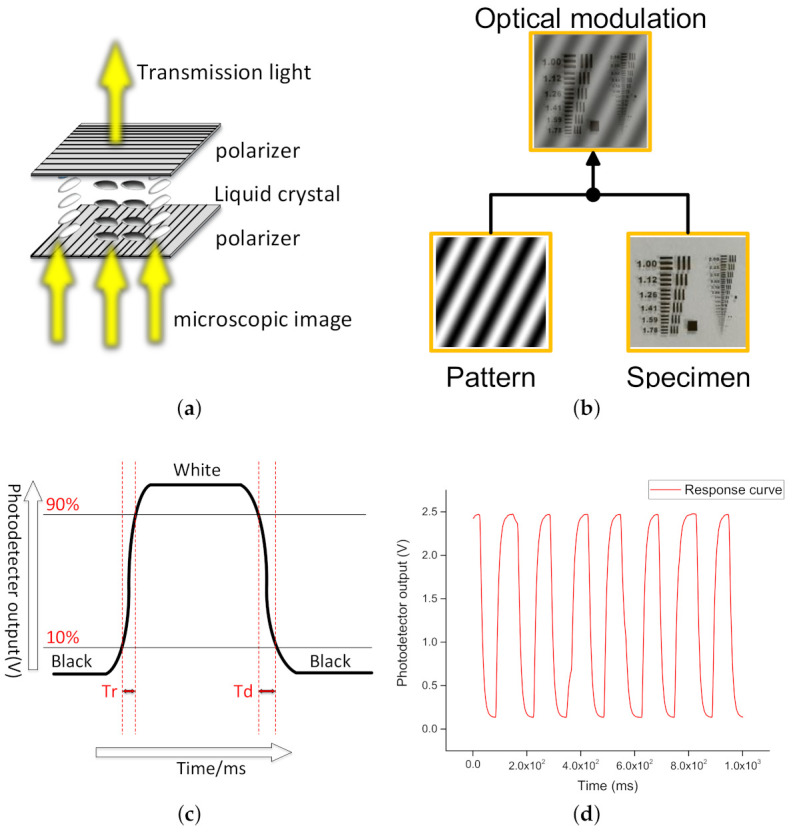
(**a**,**b**) liquid crystal modulation principle and modulation process of sinusoidal patterns as the microscopic image passes through liquid crystal spatial light modulator (LC-SLM). (**c**,**d**) the response time of the liquid crystal. The response time of LC-SLM (TSLM017-A) is Tr + Td ≤ 35 ms. The actual data of response time are measured by Data Acquisition (DAQ). The frame rate of LC-SLM is about 15 Hz. The sampling rate of DAQ is 1000 Hz.

At present, the gray response time of liquid crystal is reduced to the order of millisecond. The TSLM017-A is chose as LC-SLM to generate sinusoidal patterns (1024 Pixel (H) × 768 Pixel (V), 36.8 (H) × 27.6 mm (V)). Tr is the time for precisely twisting the liquid crystal by 90°. Td is the time for liquid crystal restoring to an undistorted state. The maximum gray-scale response time Tr + Td is 35 ms for the liquid crystal switching between 10% maximum grayscale and 90% maximum grayscale. The actual response curve is measured by the PD and recorded by the desktop multimeter. The sampling rate of the desktop multimeter is 1000 Hz. As shown in Figure 1d, the response curve of the liquid crystal is recorded. The horizontal axis shows 1000 measurements recorded in 1 s. The response curve is recorded rightly when 15 patterns including white and black patterns are displayed alternately in LC-SLM. To reduce measurement error, the LC-SLM is operated stably by 10 Hz in the latter experiments. According to Equation (1), the required sinusoidal patterns are generated by Matlab software. In the following experiments, sinusoidal patterns of different frequencies are displayed sequentially in LC-SLM.

The transmission of light is controlled by changing the shape of liquid crystals in LC-SLM. When penetrating the first polarizer, the light beam is modulated to be linear polarized. There will be no light passing through the second polarizer if the liquid crystal is not modulated because the polarization of the two polarizers is perpendicular to each other. After the polarization modulation of the liquid crystal, the light beam can partially penetrate LC-SLM. It should be noted that the linear polarized light beam is transmitted completely only when the liquid crystal is precisely twisted by 90° (Twisted nematic field effect). Therefore, the optical 2-D sinusoidal patterns are generated by controlling the shape of liquid crystals. Sinusoidal patterns of various frequencies are displayed on LC-SLM to measure the frequency domain data of the recovered object. In the experiment, LC-SLM plays pre-made sinusoidal patterns in video mode under the control of the computer. The light beam is modulated by sinusoidal 2-D patterns when penetrating LC-SLM.

**Figure 2:**
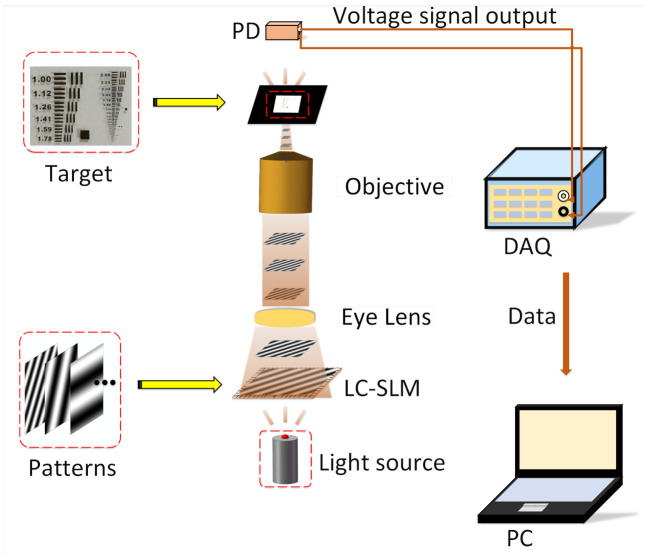
Experimental principle of transmissive single-pixel microscopic imaging. Target, USAF-1951 resolution test target.

As is shown in Figure 2, the structure of the proposed transmissive single-pixel microscope is achieved with the LC-SLM (composed of a vertical polarization filter, a horizontal polarization filter, and liquid crystals in the middle). By twisting liquid crystals with different degrees, sinusoidal patterns of different spatial frequencies are continuously generated by LC-SLM under the control of the PC. First, LC-SLM is illuminated by the light source, and patterns generated by LC-SLM are projected to the objective. When passing through the objective, patterns are zoomed out to modulate the microscopic target. Subsequently, the total light intensity is measured by the PD and then collected by DAQ. Eventually, the spatial transmittance distribution of the object is obtained by the IFFT algorithm.

## 3. Results

### 3.1. Compressed Sampling

Most of the existing microscopes are based on the array sensor to receive the light intensity signal. Large amounts of data are measured by the multiple pixel array sensor in high-resolution imaging. Then, the measured data are compressed to a Joint Picture Group (JPG) image to alleviate the storage pressure. Anyway, the redundant data are measured necessarily in the traditional microscopes. Here, the single-pixel microscope is proposed to decrease redundant measurements. Through encoding spatial information in the temporal dimension, the proposed single-pixel microscope has the property that measurement and imaging are separate. By illuminating the object with different sparse patterns, compression sampling can be achieved when measuring the sequence signal. In the obtained sequence signal, the low-frequency data represent the main information of the image while the high-frequency data represents the secondary information of the image. The microscopic image is recovered by processing the acquired sequence signals. As a result, an acceptable image can be recovered from a small number of measurements. In the experiment, to acquire the acceptable image with compressed measurements, single-pixel imaging with the compressed method first samples the main information of the image, and then gradually samples the secondary information.

**Figure 3:**
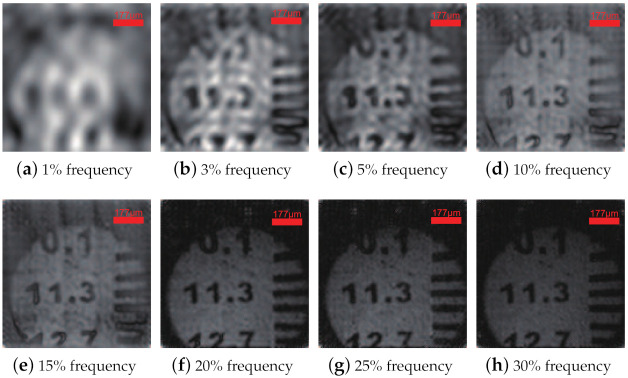
(**a**–**h**) Microimaging of different Fourier spectral sampling rates for USAF-1951 test target. The number 11.3 represents 11.3 LP/mm. Objective lens is 20× and eye lens is 5×.

Here, Fourier patterns of different frequencies are used as sparse patterns to modulate the object. To observe recovered images under different Fourier sampling rates, microscopic imaging experiments are performed with spectral sampling rates of 1%, 3%, 5%, 10%, 15%, 20%, 25%, and 30%. Besides, the magnification of the microscope maintains a constant of 100×. The LC-SLM is used to generate sinusoidal patterns of 128 × 128 at 10 Hz, and the DAQ acquires the voltage change of the PD at 1000 Hz. Each frequency in the image is sampled 100 times, and random noise interference is reduced by averaging 100 data. Only half the spectrum data need to be measured because the Fourier spectrum distribution is conjugate symmetrical. As a result, the number of sinusoidal patterns generated on the LC-SLM is reduced by half. Figure 3 shows reconstructed microscopic images at different sampling rates. The number 11.3 indicates 11.3 LP/mm, and the corresponding line width is 44 ± 1 μm. When the sampling ratio reaches 10%, the PSNR is about 17 and the SSIM is about 0.4, and the image quality remarkably improves [32]. We find that only 10% Fourier-spectra is enough for distinguishing engraving lines. Both compressed sampling and acceptable imaging are achieved in the proposed microscope.

### 3.2. High Resolution Microscopic Imaging

To demonstrate the proposed single-pixel microscope, single-pixel microscopic imaging with different magnifications is performed. The magnification of the objective determines the resolution of the microscope. The objective lens has a maximum magnification of 40×, and the corresponding numerical aperture (N.A.) is 0.65. According to the formula: $D=\frac{0.61\lambda }{\mathrm{nsin}\alpha }$, the corresponding maximum optical resolution D is 0.469 μm (assuming the average wavelength of the light source is λ = 500 nm). The USAF-1951 resolution test target (new standard) has a resolution range of 1.8 to 500 μm with an error of ±1 μm. The USAF-1951 resolution test target is employed as a specimen for quantitative evaluation of microscopic imaging resolution at different magnifications. The number δ represents the resolution of the test card. The larger number represents the higher the microscopic imaging resolution Δ: Δ=1000/2δμm; 5.04 LP/mm engraving line, 11.3 LP/mm engraving line, and 57.0 LP/mm engraving line are used for 50, 100, and 200 times magnification, respectively.

**Figure 4:**
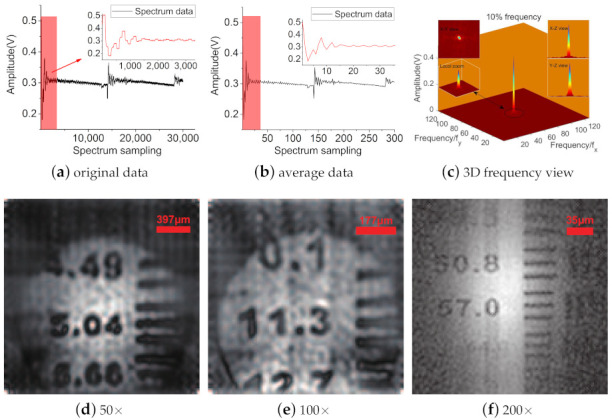
(**a**) The measurement data of Fourier spectrum. (**b**) The average value of measurement data of (**a**). (**c**) Three-dimensional visualizations of sampled spectral data. (**d**) The objective lens is 10× and the eye lens is 5×. The line width corresponding to the number 5.04 is 99 ± 1 μm. (**e**) The objective lens is 20× and the eye lens is 5×. The line width corresponding to the number 11.3 is 44 ± 1 μm. (**f**) The objective lens is 40× and the eye lens is 5×. The line width corresponding to the number 57.0 is 9 ± 1 μm.

The pixel size of the recovered microscopic image by the Fourier single-pixel imaging method is the same as the pixel size of the sparse sinusoidal pattern (128 × 128) generated by the LC-SLM. Different magnification microscopic imaging experiments were conducted at a 10% spectral sampling rate. Figure 4a is the measured sequence signal that represents the partial raw spectrum. Figure 4b shows the spectrum data after data processing and eliminating random noise. As shown in Figure 4c, the three-dimensional visualization of spectrum data demonstrates that the main information of the image is in the low-frequency part. The microscopic image is reconstructed by IFFT calculation. Figure 4d–f show images of the USAF-1951 target at different magnifications with sizes of 128 × 128. The corresponding lines on the right can be distinguished.

### 3.3. Multi-Spectral Test

The absorption spectrum of cancer cells is different from that of normal cells. Through multi-spectral microscopic imaging, the location of the cancer is easier to identify. To demonstrate the ability to detect cancer, multi-spectral imaging of cancer is performed with the proposed transmissive single-pixel microscope. In the experiments, the magnification of the microscope: the objective lens is 10×, the eyepiece is 5×. The size of Fourier patterns is 128 × 128. The human pathological section (liver cancer) was used as a biological sample and is stained with hematoxylin-eosin. The size of the sample is 2 × 2 × 0.3 cm. Here, the texture of the biological section is delicate, so 100% Fourier-spectra were measured for acquiring a high-quality image. The single-pixel imaging of the liver cancer section was performed with different optical spectra: white light (440–670 nm), red light (621 nm), and blue light (451 nm). The contrast ratio of recovered images is calculated as [48,49,50]:

$$C=\frac{\sum_{i=1}^{N}\sum_{j=1}^{8}{\left(r_{i}-r_{ij}\right)}^{2}}{M}$$

**Figure 5:**
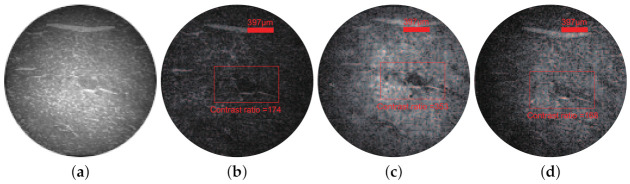
Single-pixel multi-spectral microscopic imaging of liver cancer section. (**a**) The sample image photographed by Charge Coupled Device (CCD) under white light illumination. (**b**–**d**) Single-pixel microscopic imaging under white light (440–670 nm), red light (621 nm) and blue light (451 nm) respectively.

*C* is the contrast ratio. $r_{i}$ is the grayscale value of the pixel point and $r_{ij}$ is an adjacent point of $r_{i}$; *M* is a count of the sum of squares. As shown in Figure 5b–d, dark areas in the red rectangle are the cancer nests. The contrast ratios of the red rectangle areas are 174, 353, and 168, respectively. We find that it is easier to identify cancer nests under red-light illumination compared to white-light and blue-light illumination. In the diagnosis of disease, multi-spectral microscopic imaging has the ability of multi-dimensional imaging. Compared with traditional microscopic imaging under natural light and specific wavelength, multi-spectral imaging has obvious advantages in accurately and efficiently identifying the location of the disease. Therefore, the single-pixel microscope is helpful to detect cancer through multi-spectral imaging compared with observing directly.

### 3.4. Microscopic Imaging through Scatter Media

**Figure 6:**
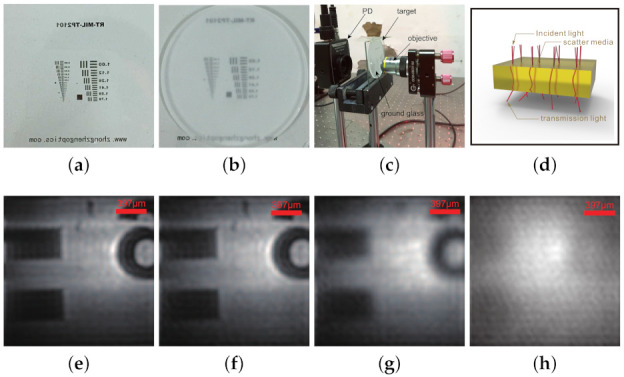
(**a**) The target to be recovered. (**b**) The ground glass diffuser. (**c**) The photograph of the optical structure. (**d**) The scatter model of the ground glass diffuser. (**e**) Image of the target without the scattering media. (**f**) Image of the target when the scattering media was between the target and the photodetector (PD). (**g**) Image of the target when the 1 mm thickness scattering media was between the target and the objective. (**h**) Image of the target when the 2 mm thickness scattering media was between the target and the objective.

The proposed single-pixel microscope is validated in microscopic imaging through scattering media. Here, the ground glass diffuser (DG20-600, N-BK7, 2 mm thickness, one side is ground glass and the other side is smooth) was used as scattering media. The USAF-1951 resolution test target was used as the object to be recovered. In the first case, the ground glass diffuser was placed between the target and PD. The light beam was scattered before being measured by PD. However, the modulation between the pattern generated by LC-SLM and the target is unaffected by the scattering media. The recovered image is shown in Figure 6f. Meanwhile, the experiment without scattering media was conducted, and the recovered image is shown in Figure 6e. Through comparing recovered images, the proposed single-pixel microscope is insensitive to the scattering media between the target and PD. In the second case, the ground glass diffuser was placed between the target and the objective. The pattern was scattered by the diffuser before being projected to the target. The pattern is blurred worse as scattering media thickness increased. When the ground glass side was close to the target, the scatter media thickness of the diffuser was about 1 mm. The recovered image of the target is shown in Figure 6g. When the smooth side was close to the target, the scattering media thickness was about 2 mm. The recovered image of the target is shown in Figure 6h. The recovered images indicate proposed microscope is sensitive to the scattering media between the target and the objective. The target is recovered acceptably when the scattering media thickness is less than 1 mm.

## 4. Discussion

We have shown that the transmissive single-pixel microscope has a simplified optical structure by utilizing LC-SLM for optical modulation. Comparing microscopic experiments with different sampling rates, the image quality under 10% of the compressed sampling, achieving PSNR = 17 and SSIM = 0.4, is enough for acceptable imaging. Through multi-spectral microscopic imaging of pathological sections, the specific location of diseased cells is easily identified. The proposed microscope is demonstrated in imaging through scattering media. Compared to traditional optical microscopes, the transmissive single-pixel microscope functions better than traditional approaches in terms of compressed sampling, multi-spectral imaging, and imaging through scattering media.

We find that the relationship between sampling rate and image quality is not linear. When the sampling rate is small, the quality of the microscopic image can be improved obviously by increasing the sampling rate. However, as the sampling rate increases, the efficiency of improving the image quality decreases fast. The optimized sampling rate could be determined based on the results of single-pixel microscopic imaging. The experimental results show that we have achieved microscopic imaging with a maximum precision of up to 9 μm. Based on the transmissive optical modulation of LC-SLM and the measurements of FFT spectrum data, the image quality of the transmissive single-pixel microscope is greatly improved, compared with the image accuracy in the single-pixel microscope based on reflective optical path and traditional reconstruction algorithm [45], which is about 30 μm.

The microscope behaves better when the scattering media is placed between target and PD. When the scattering media are placed between the target and the objective, the quality of the image will decrease significantly, the reason of which may be that the degree of the blurred pattern determined the final reconstructed image. Anyway, this is the first attempt at a single-pixel microscope using the transmissive optical path in scattering media imaging compared to the research of the reflective single-pixel microscope [25,44,45,46,51,52,53]. Moreover, we find that random noise could not be ignored or eliminated in the measurement of high-frequency data, which results in ineffective improvement of image quality even in the case of a large sampling rate. Therefore, the proposed microscope is not suitable for imaging with a large sampling rate due to the measuring error of high-frequency data caused by random noise.

## 5. Conclusions

We have come up with a single-pixel multi-spectral microscope with transmissive liquid crystal modulation, in which partially transparent Fourier patterns are displayed to modulate the object. The proposed single-pixel microscope achieves a transmissive optical system using a partially transparent LC-SLM, which simplifies the optical path of single-pixel microscopic imaging, where no complex optical path is required to project the Fourier patterns and no 4F Fourier filter is required to remove the diffraction phenomenon. Through microscopic imaging with different sampling rates, it is found that 10% Fourier-spectral reconstruction can achieve acceptable imaging quality and therefore reduce the number of measurements. Through different wavelengths of illumination, the multi-spectral images of the cancer are obtained and evaluated by the contrast ratio. Taking ground glass diffusers as scattering media, the thickness and position of the scattering media were analyzed in microscopic imaging. The single-pixel multi-spectral microscope based on transmissive liquid crystal modulation is simple and flexible, and is expected to be widely used in computational microscopic imaging and multi-modal microscopic imaging.

## Data Availability

The data and figures are available at request from the authors.

## Abbreviations

The following abbreviations are used in this manuscript:

LC-SLM	Liquid Crystal Spatial Light Modulator
CCD	Charge Coupled Device
PD	Photodetector
FT	Fourier Transform
DAQ	Data Acquisition

## References

[1] Khan M.A.U., Khan T.M., Aziz K.I., Ahmad S.S., Mir N., Elbakush E. The Use of Fourier Phase Symmetry for Thin Vessel Detection in Retinal Fundus Images. Proceedings of the 2019 IEEE International Symposium on Signal Processing and Information Technology (ISSPIT).

[2] Zheng T., Zhu H., Yao K., Pan L., Weiwei F. Design and Simulation of Optical System for Dual-wavelength Retinal oximeter. Proceedings of the 2019 IEEE International Conference on Power, Intelligent Computing and Systems (ICPICS).

[3] Truitt P.W., Magotra N., Soliz P. Spectral imaging of the human ocular fundus. Proceedings of the First Joint BMES/EMBS Conference, 1999 IEEE Engineering in Medicine and Biology 21st Annual Conference and the 1999 Annual Fall Meeting of the Biomedical Engineering Society (Cat. N, 1999).

[4] Palczewska G., Dong Z., Golczak M., Hunter J.J., Williams D.R., Alexander N.S., Palczewski K. (2014). Noninvasive two-photon microscopy imaging of mouse retina and retinal pigment epithelium through the pupil of the eye. Nat. Med..

[5] Dutta R., Manzanera S., Gambín-Regadera A., Irles E., Tajahuerce E., Lancis J., Artal P. (2019). Single-pixel imaging of the retina through scattering media. Biomed. Opt. Express.

[6] Maslov K., Zhang H.F., Hu S., Wang L.V. (2008). Optical-resolution photoacoustic microscopy for in vivo imaging of single capillaries. Opt. Lett..

[7] Zhang H.F., Maslov K., Stoica G., Wang L.V. (2006). Functional photoacoustic microscopy for high-resolution and noninvasive in vivo imaging. Nat. Biotechnol..

[8] Jain R.K., Munn L.L., Fukumura D. (2002). Dissecting Tumour Pathophysiology Using Intravital Microscopy. Nat. Rev. Cancer.

[9] Tsilimigras M.C.B., Fodor A., Jobin C. (2017). Carcinogenesis and therapeutics: the microbiota perspective. Nat. Microbiol..

[10] Quotb A., Atashkhooei R., Magaletti S., Jayat F., Tronche C., Goechnahts J., Perchoux J. (2021). Methods and Limits for Micro Scale Blood Vessel Flow Imaging in Scattering Media by Optical Feedback Interferometry: Application to Human Skin. Sensors.

[11] Bertolotti J., van Putten E.G., Blum C., Lagendijk A., Vos W.L., Mosk A.P. (2012). Non-invasive imaging through opaque scattering layers. Nature.

[12] Ghaneizad M., Kavehvash Z., Fathi H., Ghezelghaya M.A. (2021). Incoherent Holographic Optical Phase Conjugation for Imaging Through a Scattering Medium. IEEE Trans. Instrum. Meas..

[13] Zhu S., Su K., Liu Y., Yin H., Li Z., Huang F., Chen Z., Chen W., Zhang G., Chen Y. (2015). Identification of cancerous gastric cells based on common features extracted from hyperspectral microscopic images. Biomed. Opt. Express.

[14] Minsky M. (1988). Memoir on inventing the confocal scanning microscope. Scanning.

[15] Pawley J. (2006). Handbook of Biological Confocal Microscopy.

[16] Nwaneshiudu A., Kuschal C., Sakamoto F.H., Anderson R.R., Schwarzenberger K., Young R.C. (2012). Introduction to confocal microscopy. J. Investig. Dermatol..

[17] Zipfel W.R., Williams R.M., Webb W.W. (2003). Nonlinear magic: multiphoton microscopy in the biosciences. Nat. Biotechnol..

[18] Sakdinawat A., Attwood D. (2010). Nanoscale X-ray imaging. Nat. Photonics.

[19] Mikami H., Harmon J., Kobayashi H., Hamad S., Wang Y., Iwata O., Suzuki K., Ito T., Aisaka Y., Kutsuna N. (2018). Ultrafast confocal fluorescence microscopy beyond the fluorescence lifetime limit. Optica.

[20] Florimbi G., Fabelo H., Torti E., Ortega S., Marrero-Martin M., Callico G.M., Danese G., Leporati F. (2020). Towards Real-Time Computing of Intraoperative Hyperspectral Imaging for Brain Cancer Detection Using Multi-GPU Platforms. IEEE Access.

[21] Eggeling C., Ringemann C., Medda R., Schwarzmann G., Sandhoff K., Polyakova S., Belov V.N., Hein B., von Middendorff C., Schönle A. (2009). Direct observation of the nanoscale dynamics of membrane lipids in a living cell. Nature.

[22] Hell S.W., Wichmann J. (1994). Breaking the diffraction resolution limit by stimulated emission: Stimulated-emission-depletion fluorescence microscopy. Opt. Lett..

[23] Gustafsson M.G. (2005). Nonlinear structured-illumination microscopy: wide-field fluorescence imaging with theoretically unlimited resolution. Proc. Natl. Acad. Sci. USA.

[24] Huang B., Bates M., Zhuang X. (2009). Super-resolution fluorescence microscopy. Annu. Rev. Biochem..

[25] Bian L., Suo J., Situ G., Li Z., Fan J., Chen F., Dai Q. (2016). Multispectral imaging using a single bucket detector. Sci. Rep..

[26] Khemthongcharoen N., Jolivot R., Rattanavarin S., Piyawattanametha W. (2014). Advances in imaging probes and optical microendoscopic imaging techniques for early in vivo cancer assessment. Adv. Drug Deliv. Rev..

[27] Sun M.J., Zhang J.M. (2019). Single-pixel imaging and its application in three-dimensional reconstruction: A brief review. Sensors.

[28] Rizvi S., Cao J., Zhang K., Hao Q. (2019). Improving imaging quality of real-time Fourier single-pixel imaging via deep learning. Sensors.

[29] Zhao Y., Chen Q., Sui X., Gao H. (2017). Super Resolution Imaging Based on a Dynamic Single Pixel Camera. IEEE Photonics J..

[30] Soldevila F., Durán V., Clemente P., Lancis J., Tajahuerce E. Wavefront sensing by single-pixel imaging techniques. Proceedings of the 2018 International Conference Laser Optics (ICLO).

[31] Edgar M.P., Gibson G.M., Padgett M.J. (2018). Principles and prospects for single-pixel imaging. Nat. Photonics.

[32] Zhang Z., Ma X., Zhong J. (2015). Single-pixel imaging by means of Fourier spectrum acquisition. Nat. Commun..

[33] Hu X., Zhang H., Zhao Q., Yu P., Li Y., Gong L. (2019). Single-pixel phase imaging by Fourier spectrum sampling. Appl. Phys. Lett..

[34] Li X., Qi N., Jiang S., Wang Y., Li X., Sun B. (2020). Noise Suppression in Compressive Single-Pixel Imaging. Sensors.

[35] Yu X., Yang F., Gao B., Ran J., Huang X. (2020). Deep Compressive Single Pixel Imaging by Reordering Hadamard Basis: A Comparative Study. IEEE Access.

[36] Edeler T., Ohliger K., Hussmann S., Mertins A. (2012). Super-Resolution Model for a Compressed-Sensing Measurement Setup. IEEE Trans. Instrum. Meas..

[37] Ma J., Hussaini M.Y. (2011). Extensions of Compressed Imaging: Flying Sensor, Coded Mask, and Fast Decoding. IEEE Trans. Instrum. Meas..

[38] Ma J. (2011). Improved Iterative Curvelet Thresholding for Compressed Sensing and Measurement. IEEE Trans. Instrum. Meas..

[39] Deng H., Gao X., Ma M., Yao P., Guan Q., Zhong X., Zhang J. (2019). Fourier single-pixel imaging using fewer illumination patterns. Appl. Phys. Lett..

[40] Donoho D.L. (2006). Compressed sensing. IEEE Trans. Inf. Theory.

[41] Duarte M.F., Davenport M.A., Takhar D., Laska J.N., Sun T., Kelly K.F., Baraniuk R.G. (2008). Single-pixel imaging via compressive sampling. IEEE Signal Process. Mag..

[42] Katz O., Bromberg Y., Silberberg Y. (2009). Compressive ghost imaging. Appl. Phys. Lett..

[43] Gong W., Han S. (2011). Correlated imaging in scattering media. Opt. Lett..

[44] Durán V., Soldevila F., Irles E., Clemente P., Tajahuerce E., Andrés P., Lancis J. (2015). Compressive imaging in scattering media. Opt. Express.

[45] Tajahuerce E., Durán V., Clemente P., Irles E., Soldevila F., Andrés P., Lancis J. (2014). Image transmission through dynamic scattering media by single-pixel photodetection. Opt. Express.

[46] Rodríguez A., Clemente P., Tajahuerce E., Lancis J. (2016). Dual-mode optical microscope based on single-pixel imaging. Opt. Lasers Eng..

[47] Radwell N., Mitchell K.J., Gibson G.M., Edgar M.P., Bowman R., Padgett M.J. (2014). Single-pixel infrared and visible microscope. Optica.

[48] Brenner J.F., Dew B.S., Horton J.B., King T., Neurath P.W., Selles W.D. (1976). An automated microscope for cytologic research a preliminary evaluation. J. Histochem. Cytochem..

[49] Yazdanfar S., Kenny K.B., Tasimi K., Corwin A.D., Dixon E.L., Filkins R.J. (2008). Simple and robust image-based autofocusing for digital microscopy. Opt. Express.

[50] Osibote O., Dendere R., Krishnan S., Douglas T. (2010). Automated focusing in bright-field microscopy for tuberculosis detection. J. Microsc..

[51] Yao M., Cai Z., Qiu X., Li S., Peng J., Zhong J. (2020). Full-color light-field microscopy via single-pixel imaging. Opt. Express.

[52] Lenz A., Clemente P., Climent V., Lancis J., Tajahuerce E. (2019). Imaging the optical properties of turbid media with single-pixel detection based on the Kubelka–Munk model. Opt. Lett..

[53] Jauregui-Sánchez Y., Clemente P., Lancis J., Tajahuerce E. (2019). Single-pixel imaging with Fourier filtering: Application to vision through scattering media. Opt. Lett..

